# Zooming in on chromosome dynamics

**DOI:** 10.1080/15384101.2020.1757242

**Published:** 2020-05-13

**Authors:** John K. Eykelenboom, Tomoyuki U. Tanaka

**Affiliations:** Centre for Gene Regulation and Expression, School of Life Sciences, University of Dundee, Dundee, UK

**Keywords:** Chromosome structure, chromosome dynamics, live-cell imaging, fluorescent visualization, CRISPR-Cas9 technology

## Abstract

Until recently, our understanding of chromosome organization in higher eukaryotic cells has been based on analyses of large-scale, low-resolution changes in chromosomes structure. More recently, CRISPR-Cas9 technologies have allowed us to “zoom in” and visualize specific chromosome regions in live cells so that we can begin to examine in detail the dynamics of chromosome organization in individual cells. In this review, we discuss traditional methods of chromosome locus visualization and look at how CRISPR-Cas9 gene-targeting methodologies have helped improve their application. We also describe recent developments of the CRISPR-Cas9 technology that enable visualization of specific chromosome regions without the requirement for complex genetic manipulation.

## Introduction

In the late nineteenth century, Walther Flemming first documented the broad chromosome organizational changes and the subsequent segregation of sister chromatids that occurs in mitosis [1,2]. Since then many scientists have contributed to our current understanding of how chromosomes are organized at different stages of the cell cycle. In interphase, the chromosomes are relatively decondensed and occupy specific chromosome territories [3]. At this stage, the individual chromosomes are further arranged into functional units or chromosome “loops” known as topologically associated domains [4]. This organization facilitates proper gene regulation and transcriptional programs [5–7]. This current view of interphase chromosomes has emerged over two-three decades with the first realization that interphase chromosomes were organized into large mega-base loops coming from fluorescence in situ hybridization (FISH) studies [8]. The concept of spatial association between distant chromosome regions was reinforced by careful mapping of ectopic gene-targeting events [9]. More recently detailed contact maps of individual interphase chromosomes have been derived from chromosome conformation capture studies (e.g. Hi-C) [6,10]. Hi-C methodology was originally developed to yield high-resolution snap-shot molecular maps of inter and intra chromosome contacts found across populations of cells [11,12]. More recent developments of Hi-C have addressed the variation of genome organization among different cells in a population through experiments in single cells [13–17] or by high-throughput FISH [18,19]. These approaches have greatly contributed to our understanding of global genome organization and have built a picture of how cells can group functional regions of the genome for transcriptional activation or repression. These same studies also highlight the significant variation in organization, even among cells of the same population. Yet, due to the static nature of Hi-C, and other fixed-cell studies, it remains unknown just how dynamic different organization states are, i.e. how much of the observed cell-to-cell variation could be due to dynamic motions in the organized regions. This is relevant, because while individual chromosomal loci do not tend to move long distances, they nevertheless exhibit dynamic motion at a local scale [20–22] and this could contribute to the variation in organization between fixed cells.

On top of the complexities surrounding interphase chromosome organization are challenges to understand the dramatic organizational changes associated with mitosis [2]. At this stage in the cell cycle, newly duplicated chromosomes must effectively separate and become reorganized into compacted structures. Our understanding of mitotic chromosome organization has been mostly gained by visualizing whole chromosomes [23–29], by molecular studies of proteins involved in the processes [30,31] or by mapping chromosome-chromosome interactions in populations of mitotic cells using Hi-C methods [32,33]. By generating Hi-C chromosome conformation capture maps from highly synchronized cells over a time course, it has been possible to follow the organizational changes that occur between interphase and mitosis to create a picture of the sequential changes facilitated by the condensin I and condensin II complexes [32]. Nonetheless, measurements on a static population of cells lack information about the real-time dynamics of these processes.

More recently, computer-based polymer modeling has been used to try to understand how dynamic motions of chromosomes can lead to organizational changes between interphase and mitosis. Such modeling, in which chromatin is represented by theoretical flexible polymer structures, was originally developed to help understand the organization of interphase chromosomes from FISH datasets [34]. As our understanding of proteins involved in chromosome organization and their properties has improved, polymer models have become more advanced. The latest polymer models account for and include additional factors that can influence and shape the polymer structure, such as DNA-looping protein complexes like cohesin and condensin. The latest models have also benefited from validation against highly detailed datasets from Hi-C, chromatin immunoprecipitation (ChIP) sequencing and chromatin accessibility (ATAC) sequencing [35–37]. As a result, a very dynamic model of mitotic chromosome reorganization has been presented [38,39]. These models allow us to look at changes in the whole chromosome structures as well as zooming in on changes in localized chromosome regions. However, to truly understand the mechanisms of reorganization it is of great value to similarly dissect the global chromosome changes into their constitutive small-scale changes in live cells.

Interestingly, the technology that allows us to observe individual chromosome regions in living cells has existed for two to three decades [40,41] but the lack of efficient-targeted chromosome integration methods (at least in higher eukaryotic cells) has limited our ability to systematically interrogate and observe many specific chromosome locations. The development of CRISPR-Cas9 technologies, that allow efficient gene targeting in a variety of higher eukaryotic cells, has made such experiments possible [42–44]. Together with the huge amount of data now available from Hi-C and ChIP seq studies, this has paved the way to identify and visualize specific regions of the genome in live cells. This perspective will look at the development of these technologies, discuss recent progress in understanding dynamic chromosome organization and look at what the future holds.

## Methods of visualizing chromosome loci in live cells

**Figure 1.**
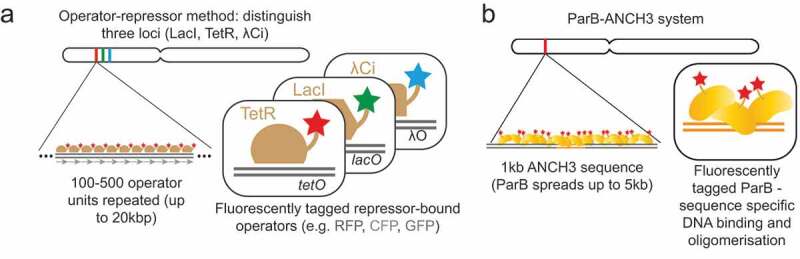
Sequence-integration methodologies for observing specific chromosomal regions in live cells by fluorescence microscopy.

Just over two decades ago the Belmont laboratory developed an elegant method to visualize chromosome regions by microscopy in live cells. This was achieved by inserting multi-copy arrays of the *E. coli lac*-operator sequence (256 repeats with total length of about 10 kbp) into the genome and expressing a fluorescently-tagged LacI repressor protein [41]. This method was further developed by the Nasmyth laboratory who exploited a distinct bacterial operator array and repressor pair, the *tet*-operator array (112 repeats with length of 6 kbp) and TetR, which allowed visualization of a second chromosome location with a different fluorescent label [40] (Figure 1(a)). Further developments of the operator array sequences, by interlacing each repeated operator sequence with a non-repetitive sequence, have meant that long operator arrays of around 12 kbp (approximately 250 repeats) are more easily obtained and stably propagated [45]. Over many years these technologies have allowed visualization of *lac*- and *tet*-operator arrays using time-lapse microscopy over long periods of time or with high temporal resolution.

Related to the *tet*- and *lac-*operator based methods, the Bystricky laboratory has developed two additional methods. The bacteriophage lambda repressor operator system works with the same principle as the *lac* and *tet* methods (Figure 1(a)) but allows simultaneous visualization of a third locus in individual cells [46]. The ParB method involves recruitment of the bacterial ParB protein to a 1 kbp non-repetitive bacterial DNA sequence, named ANCH3 (Figure 1(b)), in a sequence specific manner, followed by oligomerization of the ParB protein for up to 2 kbp from the inserted ANCH3 sequence [47]. The ParB is an attractive alternative to the operator array methods as it relies on the targeted integration of a short non-repetitive sequence to achieve similar visualization capabilities as the long (6–12 kbp) repetitive operator arrays.

These technologies have proved to be very useful in visualizing and tracking specific chromosome regions in genetically tractable model organisms such as *S. cerevisiae* and *E. coli*, allowing us to understand the active mechanisms of chromosome replication and segregation in *E. coli* [45,48] as well as the dynamics of DNA break repair [49]. In yeast, visualization of these arrays have revealed dynamic behaviors of chromosomes during interphase, including during DNA replication or following DNA damage [21,50–53]. They have also been instrumental in understanding the basis for sister chromatid cohesion following replication [40,54] and have formed the basis for studying mechanisms of chromosome capture by microtubules in early mitosis [55–58] as well as chromosome segregation at anaphase [59,60].

Many of the experiments mentioned above required targeted insertion of the *lac-* and *tet-* operator arrays at specific genomic locations in order to test different hypotheses. Since yeast and *E. coli* show high rates of homologous recombination, these organisms offered the chance for rapid, accurate integration of operator arrays in a variety of different genetic backgrounds but this was not possible in mammalian cells. Before CRISPR-Cas9 gene-targeting technology became widely available for genome editing, studies in mammalian cells mainly relied on visualization of single arrays integrated at random chromosome locations. In spite of this limitation, operator array-based studies have led to a good understanding of general chromosome dynamics during replication or transcription in mammalian cells [61–63] or during different stages of the cell cycle in Drosophila cells [64,65]. Studies have also described the effect of nuclear position or cell cycle stage on chromosome motion [20,66] and by coupling fluorescently-labeled LacI with an inducible transcriptional activator, it was determined how chromosome nuclear positioning could be altered following transcriptional activation [61]. The arrays have also been observed in mitotic chromosomes to understand regulation of mitotic chromosome stiffness by condensin complexes [67] and to visualize local chromosome dynamics during the formation and repair of chromosomal DNA double-strand breaks [68,69].

Two alternative methods, that stochastically label specific sets of chromosome regions for live visualization, have also been developed. The first one allows incorporation of fluorescently tagged nucleosides into actively replicating regions of the genome during S-phase [70]. The second method works by the binding of a fluorescently-tagged protein at methylated G^m6^ATC sequences. GATC sequences are not normally methylated in humans but by tethering the *E. coli* adenine methyltransferase (Dam) to the nuclear envelope a limited set of chromosome regions (those at the nuclear periphery) become fluorescently labeled [71]. Both methods label many small chromosomal regions in an individual cell, which can be analyzed in live cells over several cell cycles. While these methods are powerful to analyze dynamics of defined chromosome regions scattered over the genome, they are not suitable to analyze dynamics of particular chromosome loci since the regions observed in individual cells are almost certainly different from one cell to another.

## Use of CRISPR-Cas9 to visualize selected chromosome loci

**Figure 2.**
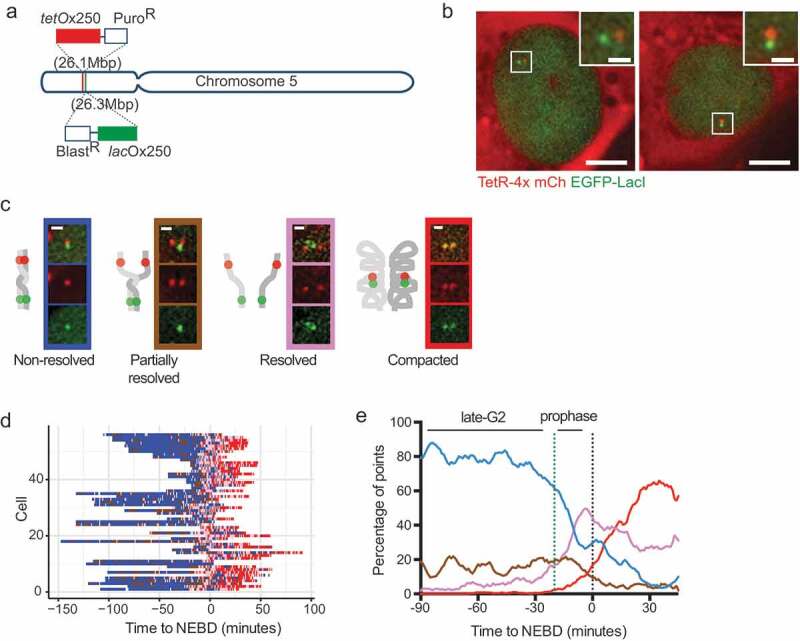
Example of fine spatial and temporal chromosome organization analyses in live cells through observation and measurement of fluorescently labeled chromosomal regions.

Sequence-specific gene-targeting methodologies – in particular CRISPR-Cas9 technology [42–44] – have opened the door to targeted genetic modification in many model organisms (including human cell lines). This has enabled targeted integration of *tet-* or *lac*-operator arrays at specific chromosomal locations (Figure 2(a)) and also means that multi-color approaches, that involve integration of two different operator arrays at two targeted loci, are a realistic proposition [72,73]. By visualizing two different colored loci, it is possible to study the dynamic behaviors within single chromosomes, or between replicated sister chromosomes – or both simultaneously (Figure 2(b–d)). Furthermore, by genetic manipulation within the localized region of the arrays, it is possible to specifically study and quantify the effect of the local chromosome landscape on the behavior within a chromosome region. In this way, in our experiments, we were able to investigate the role of a specific CTCF-binding site in organizing and maintaining sister chromatid cohesion as cells approach mitosis [72]. This observation has since been reaffirmed with a novel Hi-C methodology that can distinguish sequences from different sister chromosomes and define sister-sister chromosome contacts [74].

**Figure 3.**
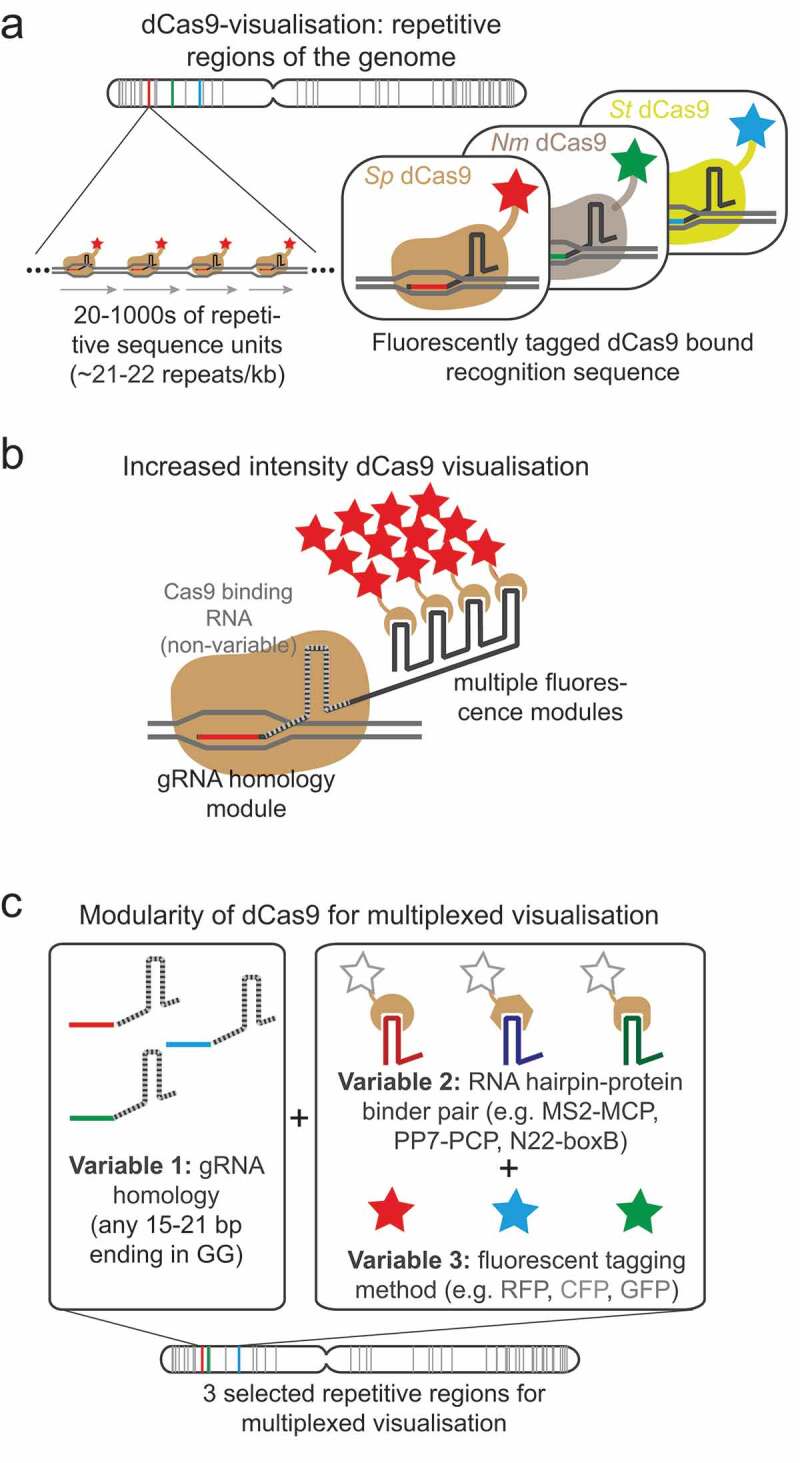
Visualizing genomic locations directly through dCas9 technologies.

Although CRISPR-Cas9 technology has made the integration of operator arrays at specific loci possible, the generation of such stable cell lines has been a time-consuming process, particularly when considering dual labeling of a particular chromosomal region. However, recent developments in the CRISPR-Cas9 field now allow imaging of a variety of genomic locations without the need for any specific genetic modification. Shortly after the advent of CRISPR-Cas9 technology it was determined that a nuclease deficient version of Cas9 (dCas9) could still bind to DNA sequences specified by the small guide (sg)RNA component [Chen, 75-77] and by fluorescently-tagging the dCas9 protein, researchers were able to visualize this binding in live cells (Figure 3(a)) [Chen, 75,78,79]. Using this method, different genomic locations can be visualized through the expression of different sgRNA(s). In order to accumulate enough fluorescent molecules at a localized chromosome region, the majority of the loci that have been successfully studied to date consist of repetitive DNA sequences that contain multiple dCas9-sgRNA binding sites in close proximity. In one study, successful visualization of a chromosome locus was achieved with just 26 binding sites [Chen, 75]. Two further studies systematically analyzed many repetitive sequences and found that the average visible locus had 41 repeats [79,80]. The development of such methods for visualizing repetitive sequences, has revealed the possibility of systematic high-throughput analyses of multiple different chromosome regions. In one high-throughput study, the cell-cycle timing of sister chromosome resolution for 16 different regions across the genome was determined. Since the selected regions were found within a variety of different chromatin landscapes, it was possible to measure the influence of different chromatin features (e.g. cohesin occupation, replication timing, transcriptional activity) on the timing of sister chromosome resolution [79]. Furthermore, by looking in live cells at the behavior of the dCas9-decorated regions [79,81] or *lac* and *tet*-operator arrays bound by their repressors [72], the process of early sister chromosome resolution in late G2 phase has been observed as a dynamic and cyclical process.

At a similar time to the development of dCas9 visualization methods, a second “gene-targeting” technology was also repurposed to visualize specific repetitive DNA. In these studies transcription activator-like effectors (TALEs), that also seek and bind specific DNA sequences, were tagged with fluorescent proteins and expressed in cells to visualize different loci [82,83]. Although this technology has been superseded by CRISPR-Cas9 visualization technology, whose target sequences are defined simply by conventional nucleotide base-pairing, this could still prove a valuable alternative. Since the mechanism of DNA binding by TALEs is through recognition of the double-stranded DNA structure, unlike the dCas9 methodology it does not involve unwinding and formation of DNA-RNA hybrid structures (R-loops). Whilst the formation of R-loops have important roles in the cell, such as in gene regulation, they can also have detrimental effects, for example, interfering with DNA replication [84]. Indeed, it has recently been reported that R-loop formation by dCas9 can lead to mutagenesis at its target site in yeast [85]. Although the possible undesired “on-target” effects of dCas9-sgRNA or TALE binding remain to be thoroughly tested and compared, it is possible that each of the methodologies might be differently suited for particular applications.

While dCas9 loci visualization technology is a versatile method, in its original iteration it cannot unambiguously distinguish different loci in the same cell since all dCas9 molecules are the same color. A number of further developments have looked to widen its applications by allowing multi-color loci visualization, for example, differential fluorescent-tagging of dCas9 proteins from multiple bacterial species was used to distinguish different loci in the same cell (Figure 3(a)) [76]. A second multiplexing method has been developed that works by modifying the sgRNA molecule through addition of functional RNA hairpin moieties (such as boxB, PP7, MS2) that bind specific proteins (N22p, PCP, MCP) that themselves are tagged with different fluorescent labels [80,86–89] (Figure 3(b,c)). Alternatively, another method that delivers pre-assembled dCas9/fluorophore-sgRNA protein complexes directly into cells has successfully been developed [90]. This methodology is useful since, unlike some of the other methods discussed, it bypasses the requirement to establish stable cell lines with optimized expression of multiple factors. Together this technology has been used to visualize and characterize motions of individual chromosomal regions during the cell cycle [81] and to examine the dynamics of non-homologous inter-chromosome contacts [87] as well as the dynamics of DNA break repair [90].

One minor limitation on the DNA sequences that are “visible” by the commonly used *Streptococcus pyogenes* (*Sp*)Cas9 is the specificity imposed by the protospacer adjacent motif (PAM). This limits standard *Sp*Cas9 to recognition of sequences that end in nGG. However, with the possibility of using Cas9 from other species that have different PAM specifications [76] or using defined mutations of *Sp*Cas9 that alter the PAM specificity [91] a wider range of sequences could be revealed. At the moment, the major limitation to dCas9 loci visualization technology is the reliance on naturally repetitive elements which are relatively rare in the genome, and hence currently the majority of chromosome regions are invisible to this methodology [http://genome.ucf.edu/CRISPRbar/ see [80]]. There is also a non-uniform distribution of repetitive sequences across the genome with the majority being found close to the chromosome ends. In order to circumvent this problem, and extend the range of visible chromosome regions, attempts have been made to increase the fluorescence intensity at each recruited dCas9 [78,80,89]. Most significantly this has been achieved in systems that use RNA hairpin binding moieties to recruit fluorescence proteins. By increasing the number of RNA hairpins on each sgRNA molecule (Figure 3(c)) the number of fluorescent molecules recruited per dCas9 complex can be increased [80]. Increased brightness has also been achieved by using brighter fluorescent protein tags, or by replacing these with a Halotag that can be covalently bound by brighter, more stable fluorophores of varying colors [80,92]. With these improvements, the minimum number of guide RNA binding sites required for visualization is reported between 4 and 20 [80,87,89]. This allows the possibility of expressing multiple closely spaced, non-repetitive sgRNAs to achieve visualization of non-repetitive regions [78]. With the current streamlined methods for cloning sgRNA molecules into multi-hairpin vectors developed in the Pederson laboratory, the generation of multiple sgRNA expression vectors should be feasible [86]. At the moment each sgRNA sequence is empirically tested to ascertain if it can be used for visualization and that it binds only to the specific target, but as our understanding of CRISPR-Cas9 site recognition improves the ability to predict and design optimal targeting sequences for any given region is likely to improve also.

## Perspective on the study of chromosome dynamics

With the development of CRISPR-Cas9 technologies that can allow live visualization of many chromosomal regions in a high-throughput manner, along with the plethora of genome-wide information describing chromosome organization in fixed-cell populations, the future of this field promises to be exciting. By using Hi-C and ChIP seq datasets [93] specific hypotheses can be developed and tested through live-cell imaging of specific genomic regions. One outstanding question in the field is regarding the origin of the variation in the structural organization of chromosomes observed among cells. It is evident, from single-cell Hi-C and FISH-based distance interrogation methods, that there is large variation in chromosome organization between cells [13,15–19] as well as through different phases of the cell cycle [14,32] and at different developmental stages [94–96]. It will be fascinating to observe and measure chromosomal organization stability in live cells and understand how much dynamic behaviors account for cell-cell variation and on what time scales these behaviors occur.

Ongoing developments in CRISPR-Cas9 technologies will also allow us to relocate chromosome regions to different nuclear compartments [97] or to stimulate/repress transcription [98,99]. Since it is possible to locate species-specific Cas9 proteins at distinct chromosome sites in the same cell [76], one can multiplex the different systems to both manipulate chromosome dynamics and visualize the behavior of particular chromosome regions of interest. Such information about chromosome dynamics under different situations, and derived over different time-scales, will be of great use for fine-tuning mathematical polymer models of chromosome organization [35–39] which will help us to gain a deeper understanding of the basis of chromosome organization.

We are now in an era where huge amounts of data exist that describe both the physical organization of the DNA in our genomes as well as its protein make-up in the form of the overlying chromatin landscape. With imaging technology improving and the development of the techniques for observing specific chromosomal regions in live cells, we now enter a period where we can address the relationships between detailed organization regimens and the dynamic processes that establish or maintain them. Since chromosome organization is important for many fundamental processes that are intricately linked to human diseases such as cancer (including maintenance of proper transcriptional control, chromosome segregation at mitosis or DNA repair), a deeper understanding of mechanisms behind these processes will lead to a better understanding of human diseases. Ultimately, enhancing our understanding may uncover potential new targets that can be exploited for treatment of such diseases.
